# Key performance indicators in pre-hospital response to disasters and mass casualty incidents: a scoping review

**DOI:** 10.1007/s00068-024-02533-8

**Published:** 2024-07-11

**Authors:** Nikolaos Markou-Pappas, Hamdi Lamine, Luca Ragazzoni, Marta Caviglia

**Affiliations:** 1 CRIMEDIM – Center for Research and Training in Disaster Medicine, Humanitarian Aid and Global Health, Università del Piemonte Orientale, via Bernardino Lanino, 1, 28100 Novara, Italy; 2 Department of Translational Medicine, Università del Piemonte Orientale, 28100 Novara, Italy; 3 Department for Sustainable Development and Ecological Transition, Università del Piemonte Orientale, 13100 Vercelli, Italy

**Keywords:** Review, Disaster response, Mass casualty incident, Pre-hospital care, Performance evaluation, Key performance indicators

## Abstract

**Purpose:**

The objective of this study was to offer a comprehensive synthesis of the existing Key performance indicators (KPIs) used in the evaluation of the pre-Hospital response to disasters and mass casualty incidents (MCIs).

**Methods:**

At the end of December 2022 a scoping review has been performed on PubMed, Scopus, Embase, and Medline to identify articles describing the use of KPIs to assess the performance of first responders during the prehospital phase of an MCI (real or simulated). Following the Preferred Reporting Items for Systematic Reviews and Meta-Analyses guidelines, fourteen articles were included in the analysis.

**Results:**

Eleven articles applied indicators in exercises and/or simulations. Two articles proposed new KPIs, and one used KPIs for developing a model for benchmarking pre-Hospital response. All articles analyzed quantitative indicators of time, whereas two studied indicators of structure, of process, and of outcome as well.

**Conclusion:**

The findings from this review emphasize the need for employing common terminology and using uniformed data collection tools, if obtaining standardized evaluation method is the goal to be achieved.

**Supplementary Information:**

The online version contains supplementary material available at 10.1007/s00068-024-02533-8.

## Introduction

Disasters are commonly defined as any event causing a *“serious disruption of the functioning of a community or a society involving widespread human, material, economic or environmental losses and impacts, which exceeds the ability of the affected community or society to cope using its own resources”.* Due to climate change, escalating migration and refugee crises, outbreaks of epidemics and pandemics, and civilian casualties in contemporary conflicts around the world, disasters are occurring more frequently putting more people at peril [1, 2]. Along with their frequency, the nature of disasters is changing as well. They are becoming more complex, unpredictable, and prolonged [3], thus calling for advancement in disaster management to bolster preparedness and fortify resilience against future calamities [4], as strongly advocated by the Sendai Framework for Disaster Risk Reduction 2015–2030 [2]. Disasters, induce among other consequences what in the literature is defined as mass casualty incidents (MCIs) [5]. The timely, effective, and efficient response of emergency medical services (EMS) personnel is crucial in mitigating the immediate human impact of MCIs [6], as well as in minimizing the risk of both short-term and long-term complications, and facilitating a prompt and effective management of the event, from the incident site (i.e. prehospital) to healthcare facilities [7, 8]. Nonetheless, such response has seldom been evaluated [9]. Identifying areas of improvement can enhance overall disaster health management and can strengthen the level of preparedness of a health system [10, 11], by allowing decision-makers to make well-informed decisions [12]. It can also help increase the quality of services during the response to a disaster in real life, by inducing an implementation of higher quality of education and training provided to the first responders [13].

Employing key performance indicators (KPIs) that concentrate on the evaluation of the response to an MCI, including the performance of first responders in the prehospital phase both in simulated and real events, has been useful for addressing such lack of evaluation [14].

According to the Oxford’s dictionary, a KPI is defined as a measurement of an individual’s, a team’s, or a department’s achievement, and its development is a part of a performance management system [15]. These indicators may be quantitative or qualitative depending on what they are evaluating, and the unit of measure used to express such evaluation [16]. Quantitative KPIs can be divided in two categories: of time and of structure. The temporal performance indicators use seconds, minutes or even hours as a unit, whereas the indicators of structure use multiples of whole positive numbers to describe how many units of items (an ambulance, a doctor, or a tabard) may be needed [17]. On the other hand, under the purview of the qualitative KPIs, all those indicators that assess a process unfolding in a certain specific sequence of events, or an outcome can be found [17].

Even though leading experts in the field of Disaster Medicine are continuously addressing the issue of establishing standards that may be used as templates for evaluation and research, there is currently no agreement on criteria for indicators that can be used as a tool for quality control and to assess performance in major incidents. Indeed, when a response is not evaluated using predetermined, high-quality data, it cannot be utilized for analysis, comparison, experience sharing, inter-agency cooperation, and the advancement of scientific methodology [18, 19]. The aim of this scoping review is therefore to offer a comprehensive synthesis of the existing KPIs used in the evaluation of the prehospital response to disasters and MCIs (real or simulated).

## Methods

### Search strategy and selection criteria

A systematic literature search was performed on PubMed, Scopus, Embase, and Medline to identify articles exploring KPIs used to assess the performance of first responders during the prehospital phase of an MCI (real or simulated). The search strategy concentrated on papers published in English until December 25, 2022, using the following search terms (including synonyms): “key performance indicator”, “process assessment”, “health care quality”, “performance evaluation” AND “mass casualty incidents”, “disaster”, “health crisis” AND “prehospital care”, “acute emergency care”.

No restrictions to the time period or any filters were applied.

Peer-reviewed studies, textbooks, consensus guidelines, protocols, framework, and models were included. Exclusion criteria were non-English papers, articles that did not focus specifically on KPIs used to assess the prehospital phase during an MCI, abstract and conference papers, or unverified or unsubstantiated press and news media reports.

References and cited articles were screened for additional relevant publications, consequently included in the selection process. The Preferred Reporting Items for Systematic Reviews and Meta-Analyses (PRISMA) guideline was followed.

### Data collection and analysis

NMP and HL independently screened titles and abstracts of articles yielded by the search using the Rayyan Intelligent Systematic Review tool [20]. The same two investigators separately reviewed the full text of included articles and removed any paper that did not meet the inclusion criteria. After each phase of the screening process (based on titles/abstracts, and full text screening) a cross match of the decisions of the two investigators was made. When a conflict on whether to include or exclude one of the articles arose, it was resolved through a discussion and a final consensus was reached representing both parties. The investigators did not perform any kind of pooled analysis of the included papers due to the broad inclusion strategy, the anticipated significant heterogeneity and the paucity of literature on the subject. PRISMA and statement checklists guided the data extraction and evaluation process, following a combination of inductive and deductive approaches filtering thus the reports based on quality or bias risk. Key information such as the type of MCI, KPIs identified and related benchmarks, the elements of the prehospital phase assessed by the studied KPIs, and the approach to the studied KPIs (creation of new KPIs, test, and/or validation) were recorded through a pre-established extraction sheet. Results were discussed between authors (NMP, HL, and MC) before data analysis.

## Results

**Fig. 1:**
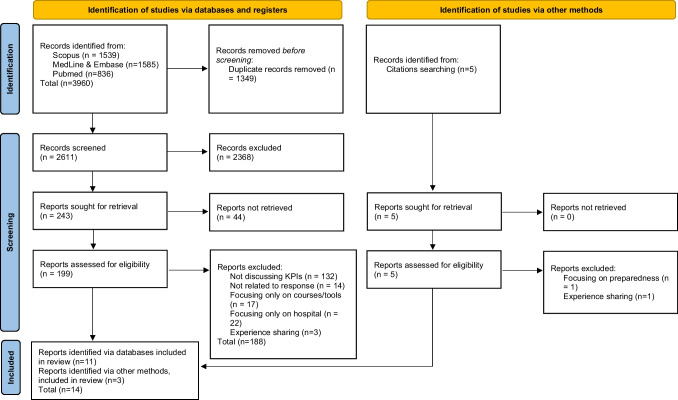
PRISMA

Of 3960 articles identified in the database search, 243 met the eligibility criteria for full text screening. After full text screening, 188 articles were excluded, while 44 articles were not available (inaccessible due to access rights, full text not available etc.), leaving 11 articles meeting the full relevant criteria. After screening citation and references, 3 additional articles were identified and included (Fig. 1).

In Table 1, the details of each paper are presented. The included articles refer to a 22-year time span (from 1997 to 2019). Of note, 6 papers focusing on almost overlapping KPIs were published by a Swedish research group, although their research encompassed different settings (Sweden [14, 19, 21–23], Afghanistan [24]). Eight included articles [16, 17, 19, 22, 24–27] concentrated on the overall prehospital management of MCIs, including different aspects from the initial scene assessment to the evacuation of casualties. Among these, indicators examining triage, prehospital treatment, evacuation (MEDEVAC) can be found (Table 1). The remaining 5 articles concentrated on distinct facets of prehospital management, such as transportation, communication, triage and command and control, the latter referring to the management and coordination of emergency response activities by an established authority or hierarchy of authorities.

**Table 1:** Synthesis of the included articles

Code	Title	Authors	Year	Country	Approach to KPIs	Elements of pre-hospital response examined
A1	Performance indicators for prehospital command and control in training of medical first responders	Anders Rüter, Per Ortenwall, Thore Wikstrom	2004	Sweden	Applying on an exercise	Command and Control
A2	Performance Indicators for Major Incident Medical Management – A Possible Tool for Quality Control?	Anders Rüter, Per Ortenwall, Thore Wikstrom	2004	Sweden	Applying on real past MCIs	Command and Control
A3	Evaluation of Medical Command and Control Using Performance Indicators in a Full-Scale, Major Aircraft Accident Exercise	Dan Gryth, Monica Radestad, Heléne Nilsson, Ola Nerf, Leif Svensson, Maaret Castrén, Anders Rüter	2010	Sweden	Applying on an exercise	Overall process
A4	Performance indicators for initial regional medical response to major incidents: a possible quality control tool	Heléne Nilsson, Tore Vikström, Carl-Oscar Jonson	2012	Sweden	Applying on real past MCIs	Command and Control
A5	Disaster Metrics: Quantitative Estimation of the Number of Ambulances Required in Trauma- Related Multiple Casualty Events	Jamil D. Bayram, Shawki Zuabi, Mazen J. El Sayed	2012	DNA^a^	Applying on an exercise	Transportation
A6	Resource planning for ambulance services in mass casualty incidents: a DES-based policy model	Marion S. Rauner & Michaela M. Schaffhauser-Linzatti, Helmut Niessner	2012	Austria	Applying on an exercise	Overall process
A7	Utstein-Style Template for Uniform Data Reporting of Acute Medical Response in Disasters	Michel Debacker, Ives Hubloue, Erwin Dhondt, Gerald Rockenschaub, Anders Rüter, Tudor Codreanu, Kristi L. Koenig, Carl Schultz, Kobi Peleg, Pinchas Halpern, Samuel Stratton, Francesco Della Corte, Herman Delooz, Pier Luigi Ingrassia, Davide Colombo, Maaret Castrèn	2012	DNA^a^	Proposing new	Overall process
A8	Combining performance and outcome indicators can be used in a standardized way: a pilot study of two multidisciplinary, full-scale major aircraft exercises	Monica Rådestad, Heléne Nilsson, Maaret Castrén, Leif Svensson, Anders Rüter and Dan Gryth	2012	Sweden	Applying on an exercise	Overall process
A9	Disaster Metrics: A Proposed Quantitative Model for Benchmarking Prehospital Medical Response in Trauma-Related Multiple Casualty Events	Jamil D. Bayram, MD, MPH, EMDM, MEd; Shawki Zuabi, MD, EMDM	2012	DNA^a^	Using for a model	Overall process
A10	Essential key indicators for disaster medical response suggested to be included in a national uniform protocol for documentation of major incidents: a Delphi study	Monica Rådestad, Maria Jirwe, Maaret Castren, Leif Svensson, Dan Gryth, Anders Rüter	2013	Sweden	Proposing new	Overall process
A11	Impact of training in medical disaster management: a pilot study using a new tool for live simulation	Pier Luigi Ingrassia, Davide Colombo, Federico Lorenzo Barra, Luca Carenzo, Jeffrey Franc, Francesco Della Corte	2014	Italy	Applying on an exercise	Overall process
A12	Performance indicators for prehospital command and control developed for civilian use tested in a military training setting, a pilot study	L Lundberg, A Jonsson, T Vikström, Andres Rüters	2015	Afghanistan	Applying on an exercise	Overall process
A13	Intuitive versus Algorithmic Triage	Alexander Hart, Elias Nammour, Virginia Mangolds, John Broach	2018	USA	Applying on an exercise	Triage
A14	Dynamic Communication Quantification Model for Measuring Information Management During Mass-Casualty Incident Simulations	Omer Perry, Eli Jaffe, Yuval Bitan	2022	DNA^a^	Applying on an exercise	Communication

^a^Data not available

Out of the 14 articles, 2 are proposing new KPIs [16, 17], 1 is using KPIs for developing a model for benchmarking prehospital response, whereas the other 11 articles [14, 19, 21–25, 27–30] applied the studied indicators through exercises and/or simulations. In these 11 articles, the responders are professionals who have had training before their involvement in the different incidents or simulations. Most commonly the responders’ teams were composed by medical doctors, registered nurses and/or trained paramedics-namely EMS personnel. In one case the participants were military personnel with similar training and in another the participants were students majoring in Health Programs, with most of them being medics at a city EMS with 2 or 3 years of military experience [30].

In Table 2, information regarding the number, the type (quantitative/qualitative) and the benchmarks (when provided) for the KPIs in each article included, is summarised. Quantitative KPIs included time indicators and indicators of structure, for which the definition “a quantitative measure reflecting availability of resources, for example number *of ambulances, involved in medical response at a major incident*” provided by Rådestad was adopted [17]. Qualitative KPIs included indicators of process and indicators of outcome. For the former, Rådestad’s definition of “*indicator describing activities or processes involved in medical response management at a major incident and is usually associated with patient outcome*” was once again adopted [17], while the latter were defined either as “*indicators describing the outcome of health care, in disaster medical care the reduction of morbidity and mortality of the survivors is the most important outcome”* [17] *or as “measures of the actual achievements intended*” [16].

**Table 2:** Distribution of quantitative and qualitative KPIs

Code	Number of KPIs	Quantitative		Qualitative		Benchmarks provided related to KPIs
		of Time	of Structure	of Process	of Outcome	
A1	11	✘		✘	✘	Yes
A2	8	✘		✘	✘	Yes
A3	23	✘		✘	✘	Yes
A4	11	✘		✘	✘	Yes
A5	9	✘	✘			Yes
A6	32	✘	✘		✘	Partially
A7	36	✘	✘	✘	✘	Partially
A8	23	✘		✘	✘	Yes
A9	15	✘				Partially
A10	59	✘	✘	✘	✘	No
A11	16	✘		✘		Partially
A12	9^b^	✘		✘	✘	Yes
A13	2	✘			✘	Yes
A14	13	✘		✘	✘	Partially

^b^The KPIs applied were 11, but 2 of them were later discarded as non-applicable in a military setting

All articles included analysed quantitative indicators of time, whereas 2 papers expanded their study in all 4 different categories of KPIs abovementioned [16, 17]. Even though the identification of the benchmarks was not the aim of our study, it is important to note that more than half of the papers analysed, included a full list of benchmarks proposed [14, 19, 21–24, 28, 29]. The authors of one article did not provide any benchmark regarding the indicators that they examined [17] (Table 2).

Out of the 268 KPIs identified, 79 are unique to the papers studying them, 22 have been mentioned twice (Annex). The rest 145 have been mentioned in three or more papers and were clustered by the authors of this review according to the area of prehospital response they address. The most frequently examined areas are the Guidelines and Management, whereas the Second and First report come up as the secondly and thirdly most frequently assessed ones (Table 3).

**Table 3:** Most frequently used KPIs (≥ 3 times)

Concept	KPIs	Frequency
Additional resources	Decision on sending additional resources to scene	**4**
	Assessment if resources in own organization are adequate	**4**
Ambulances	Time of arrival of the first EMS ambulance at the incident site	**4**
	Number of ambulance vehicles (ALS/BLS) on-site	**4**
Contact	Establishing contact with strategic level of command and control	**6**
	Establishing contact with incident officers	**3**
Declaration	Declaring major incident	**4**
First report	First report to dispatch centre from scene “Window report”	**8**
	Correct content of first report (according to METHANE)	**5**
First responders	Number of physicians on-site	**3**
	Number of rapid response teams	**3**
Guidelines	Formulate guidelines for response	**9**
	Content of the first management at the incident site, decisions about the course of action/issues guidelines for the medical response	**3**
	Time point when regional medical command centre issues guidelines for course of action	**4**
	Deciding on guidelines for referring hospitals	**9**
Injuries	The total number of injured	**3**
Liaison	Liaison with fire and police incident officers on scene	**6**
Management	Disaster medical operations coordination	**4**
	Patient access interval (from arrival at scene to arrival at patient)	**3**
	Scene treatment time interval (from beginning first intervention to beginning to move the patient)	**3**
	First patient evacuated	**6**
	Transport time interval (from leaving the scene to arrival at hospital)	**3**
Media	First information to media on scene	**9**
Medical ambition	Establishing level of medical ambition	**5**
Second report	Time of Second report from scene	**8**
	Content of second report from scene	**8**
Tabard	Putting on tabard (indicating medical and ambulance incident officer)	**6**
Triage	Time to first triage	**3**
	Under-triage and over-triage	**5**

## Discussion

This systematic review offers a comprehensive synthesis of the existing KPIs used in the evaluation of the prehospital response to disasters and MCIs. The crucial need for standardized terminology, uniform data collection tools, and established benchmarks for assessing prehospital responder performance was highlighted.

Before delving into the content of the KPIs, the geographical distribution of included work is worth mentioning. While a global distribution of studies was anticipated, the majority of included articles were produced by Swedish research teams. To the authors’ understanding, this may have been facilitated by the necessity to implement the Swedish national preparedness plan and by the presence of KAMEDO (Katastrofmedicinska Organisationskommitten), a Swedish Organisation for Studies and Reports from International Disasters organized and funded by the Swedish National Board of Health and Welfare [21]. However, this raises the question of the applicability of these KPIs in other countries with different physical geography, environments, resources, and legal and regulatory frameworks. Echoing the relevance of such question is the study performed in Afghanistan [24]. The study’s aim was to test if the performance indicators for prehospital command and control developed for civilian use can be used in a military training setting. The finding that two KPIs were deemed non-relevant is significant, as it suggests that caution should be exercised when applying the same indicators universally and without reservation, given that not all prehospital emergency care is provided in optimal conditions [31].

Another aspect of interest is that the authors of some of the included papers were able to elaborate a series of KPIs only upon examination of after-action reports of MCIs that occurred in a time span of 22 years [21]. A possible explanation of why such a long period of time had to be reviewed to produce the aforementioned KPIs could be that MCI after-action reports are typically performed for purposes other than performance evaluation. Indeed, they often simply summarize different aspects of the response, while actions and decisions taken at the operational and tactical levels are rarely registered in a thorough and complete manner, thus preventing the comprehensive identification of indicators of performance [9, 21].

When attempting to determine the most studied element of the response phase, management, formation of guidelines (for either response in general or specifically to the evacuation of patients) and communication (whether it be the first or second report) were the most frequently examined. This finding may be explained by the fact that these areas are often identified as having shortcomings. Any intervention that could improve the standardization of prehospital response to MCIs and enhance communication efficiency could have a significant impact on the success of disaster management [32].

It becomes clear from the studies covered in this review that notwithstanding the introduction of multiple frameworks to enable uniform disaster research and evaluation [33], lack in use of consistent (or any) terminology across the various phases of a disaster persists. The epitome of this issue is the use of performance indicators of time: while all authors are either applying or proposing new KPIs, only two have been proposing definitions. Such a discovery contributes to the general confusion and sets back even more the search of commonly set, accepted, and used guidelines in the response evaluation.

Although the World Association for Disaster and Emergency Medicine (WADEM) has published a policy document on evaluation and research where the question of adopting a more evidence-based to disaster medicine research is raised [34], in all 14 articles included in this review the need for further validation of the indicators studies and used, is always highlighted. That leads, though, to the point that no validated set of KPIs on which to base further research, currently exists. This observation further underscores the need to improve the science behind the development, validation, and use of indicators.

When examining the use of quantitative and qualitative KPIs, it is clear that there is a discrepancy in the number of articles focusing on the former as opposed to the latter. Specifically, despite all 14 articles studied temporal performance indicators, 10 were looking into indicators of process [14, 16, 17, 19, 21–24, 27, 30], either that is accuracy or respect of the sequence of steps of which such process is comprised. An example that could function as the embodiment of such anomaly is the first report to the dispatch centre (METHANE). The focus appears to be primarily on the timely arrival of communication, rather than the accuracy of the report's content. However, this does not necessarily indicate that communicating something earlier is more important than communicating it correctly. The authors believe that a more plausible explanation for this discrepancy is the difficulty of evaluating communication quality. While the timing of communication can be assessed using time stamps and stopwatches, properly evaluating the quality of communication requires a validated training curriculum and a validated set of KPIs. Unfortunately, the latter still seems to be out of reach [22, 29].

To conclude, upon studying the articles included in this research, the reader may find it difficult to trace the origin and rationale behind many of the proposed benchmarks. Additionally, some authors only provided benchmarking for a portion of the examined performance indicators [16, 25–27, 30]. As previously mentioned, setting a value against which individual responders or the overall system performance can be evaluated is always a challenge [21]. However, the concept of benchmarks is inherent to the usefulness of a proposed indicator as long as it is explicit that the indicators are not being used to single out failures and to identify scapegoats, but rather to identify areas where improvements can be made [21].

### Limitations

First, this review has only included articles in English. It is possible that other pertinent research in languages other than English was skipped over for this review. Secondly, a quality assessment of the included studies was not performed using a validated tool but by merely using the reviewers’ experience in research, this decision was taken because of the small number of papers that examined KPIs on the prehospital response found.

## Recommendations

The findings of this review demonstrate the pressing need to establish standards for evaluating performance to disaster response. In the spirit of satisfying such need, some recommendations based on the results and discussion section of this review, are presented below:

- According to the authors -in complete accordance with Coats statement- set, accept, and employ the same definitions and terminology is the first step towards developing a more systematic research approach in Disaster Medicine and consequently, a better system for patient care [35].
- Data must be available and preferably recorded in a way that evaluation can be performed without delays and must include all decisions made, when they were taken, and by whom.
- An attempt to validate in a scientific way the already existing in the literature KPIs should go hand in hand with the proposal of new ones. The creation of a commonly accepted, validated performance indicators will push long way the evaluation of response to a disaster.
- Measurable KPIs should be built into the training of responders in management, command and control and, overall, in the different levels of response to major incidents and disasters. Making sure that the indicators from various training programmes are compatible is not a consideration to strive for.
- In the context of an ever-changing Disaster Medicine landscape, the introduction of the term Complex Public Health Crises in 2020 mirrors the need to change not only the way we respond to disasters but the way we approach them in total [3]. Even though it may seem premature, it is the authors adamant belief that developing KPIs measuring the public health and the mental health support interventions should be a priority.

## Conclusion

This literature review systematically examines the published data on KPIs used to evaluate prehospital response during disasters and MCIs. The findings reveal that the absence of standardized terminology and inconsistent data collection methods have resulted in a limited number of KPIs. To address this issue, there is a need to establish standards for evaluating prehospital responders' performance in these situations. This includes using a common terminology, implementing structured data collection systems for both real and simulated events that cover all prehospital processes, and employing validated KPIs for proper performance evaluation. Objective and measurable data will enable experts and researchers to effectively assess and improve prehospital medical response to disasters and MCIs.

## Supplementary Information

Below is the link to the electronic supplementary material.

Supplementary file1 (DOCX 23 KB)

Supplementary file2 (PDF 988 KB)

## Data Availability

No datasets were generated or analysed during the current study.

## Declarations

The funders had no role in the design of the study; in the collection, analyses, or interpretation of data; in the writing of the manuscript; or in the decision to publish the results.

### Disclaimer

Content reflects only the authors’ view and the Research Executive Agency, and the European Commission are not responsible for any use that may be made of the information it contains.
