# Supplementary material for: Key performance indicators in pre-hospital response to disasters and mass casualty incidents: a scoping review
Source: Eur J Trauma Emerg Surg. 2024 Jul 11;50(5):2029–37. doi: 10.1007/s00068-024-02533-8 (PMC11599410; doi:10.1007/s00068-024-02533-8)
Supplement: Supplementary file 1 — Supplementary file1 (DOCX 23 KB) [file 68_2024_2533_MOESM1_ESM.docx]

| Annex | |
| --- | --- |
| KPI | **Frequency** |
| Content of first report | 2 |
| Ensuring that there is adequate information for decision on referrals | 2 |
| Delivery time interval (from arrival at hospital to transfer of care) | 2 |
| Disturbance and type of disturbance of documentation systems | 2 |
| Ensuring that there is adequate information for decision on referrals | 2 |
| Initial assessment time interval (from arrival at patient to beginning first intervention) | 2 |
| Mean utilization rate of physicians | 2 |
| Mortality on scene (number) | 2 |
| Number of mobile medical teams and time point when alarming each one | 2 |
| Number of physicians as commanders on-site | 2 |
| Patient removal time interval (from beginning to move the patient to leaving the scene) | 2 |
| Percentage of 'delayed' category (T2) survivors triaged by first responders per time unit after initiation of the response | 2 |
| Percentage of 'immediate' category (T1) survivors triaged by first responders per time unit after initiation of the response | 2 |
| Rate of individuals severely injured | 2 |
| Recovery time interval (from transfer of care to back to service) | 2 |
| The number of 'delayed' T2 category patients transported from the scene to receiving healthcare facilities | 2 |
| The number of 'immediate' T1 category patients transported from the scene to receiving healthcare facilities | 2 |
| Time point for the first referral of patients to the receiving health care facilities (referral key) | 2 |
| Time point of notification of the first appropriate staff person to assure medical management coordination role | 2 |
| Time point when on scene medical control and coordination is demobilised | 2 |
| Time point when on scene medical control and coordination is operational | 2 |
| Total rescue time | 2 |
| (Δ1) the difference between the actual number of casualties at the scene and the number of casualties that were reported to the commander | 1 |
| (Δ2) the difference between the actual number of casualties and the number of casualties that were reported as evacuated by the commander at the end of the simulation. | 1 |
| (Δ3) the time at which the commander started to acquire information about the overall number of casualties | 1 |
| (Δ4) the time at which the commander started to acquire information about the evacuation of casualties. | 1 |
| Affected but uninjured (estimated number) | 1 |
| All time points for the updated references of patients to the receiving healthcare facilities | 1 |
| Ambulance activation time interval  (from reception of the call to ambulance departure) | 1 |
| Ambulance response time interval  (from ambulance departure to arrival at scene) | 1 |
| Decision on level of alert for staff | 1 |
| Decisions on sending liaison officer to county administrative board | 1 |
| Dominating type of injury | 1 |
| Duration of individual’s injured person’s recovery | 1 |
| Duration of pre-triage per injured person | 1 |
| Establishing continuous communication with dispatch center | 1 |
| First physician arrives after x minutes at incident site | 1 |
| First rapid response team arrives after x minutes at incident site | 1 |
| Immediate category' deaths within the total population of 'immediate category' survivors | 1 |
| Incident type | 1 |
| Injury severity (ISS) | 1 |
| Instructions and verbal communications were directed | 1 |
| Instructions were explicit | 1 |
| Location of incident | 1 |
| Make sure there is information for definitive referral guidelines | 1 |
| Maximum Time Allowed T1 | 1 |
| Maximum Time Allowed T2 | 1 |
| Mean Time to Triage | 1 |
| Medical communication and information management | 1 |
| Medics needed for setup of advanced medical post | 1 |
| Mortality Pre-hospital and during transport (number) | 1 |
| Moving speed for simulated items | 1 |
| Number of ambulance helicopters alarmed and time point for alarming each | 1 |
| Number of first responder vehicles and time point for alarming each | 1 |
| On-scene initial ation by first medical responder | 1 |
| On-site stabilisation or treatment | 1 |
| Operational level of regional medical command centre | 1 |
| Patient outcome (preventable complication, preventable death) | 1 |
| Percentage of 'delayed' category survivors stabilized in a 'treatment area' per time unit after initiation of the response | 1 |
| Percentage of 'delayed' category survivors transported from the scene to receiving healthcare facilities per time unit after initiation of the response | 1 |
| Percentage of 'immediate' category survivors stabilized in a 'treatment area' per time unit after initiation of the response | 1 |
| Percentage of 'immediate' category survivors transported from the scene to receiving healthcare facilities per time unit after initiation of the response | 1 |
| Percentage of impact deaths within the total population of disaster victims | 1 |
| Performance of pre-triage | 1 |
| Possible future developments or requirements were communicated clearly | 1 |
| Pre-hospital treatment accuracy (Oxygen therapy, Open airway, Respiratory assistance, PNX detension, Haemorrhage control, Fluids, Immobilization, Analgesia) | 1 |
| Setup time for other treatment rooms | 1 |
| Setup time for treatment room I | 1 |
| Situational information was verbalized | 1 |
| Start of injured person’s recovery after x minutes | 1 |
| Systematic trigger criteria for activation of the DMMP | 1 |
| The measures initiated to address operational /infrastructure disruption | 1 |
| The medical rescue factor R | 1 |
| The number of T3 category patients transported from the scene to receiving healthcare facilities | 1 |
| The number of T4 category patients transported from the scene to receiving healthcare facilities | 1 |
| The percentage of medical staff on the call-down list who reported at the appropriate location in the predetermined time delineated in the DMMP | 1 |
| Time between arrival of ambulance vehicles | 1 |
| Time between arrival of physicians | 1 |
| Time between arrival of rapid response teams | 1 |
| Time Factor (TF) | 1 |
| Time for decision if another county / region needs to be alerted and content of this decision | 1 |
| Time point and if other person than DO assumes the role as initial medical management coordinator | 1 |
| Time point at which triage was ordered by the medical incident commander | 1 |
| Time point for established liaison with agencies and organisations affected on matters of strategic management (rescue service, police) | 1 |
| Time point of activation of the DMMP | 1 |
| Time point of incoming call at dispatch center or equivalent | 1 |
| Time point of occurrence of disaster | 1 |
| Time point of that last staff person notified has reported to the appropriate location mentioned in the DMMP | 1 |
| Time point of transportation of last ill/injured survivor from scene by EMS | 1 |
| Time point when contact made with the authority at the national level | 1 |
| Time point when liaison is established with other actors, emergency support function (ESP) | 1 |
| Time point when on scene medical management establishes contact with regional medical command centre | 1 |
| Time point when regional medical command centre establishes contact with management at the scene of the incident | 1 |
| Time points at which medical responders at scene are activated and demobilised | 1 |
| Time to first move (Collecting Area / Advanced Medical Post) | 1 |
| Time to last triage | 1 |
| Time when casualty-clearing station can start receiving patients | 1 |
| Triage system used | 1 |
| Waiting time for treatment in treatment room I | 1 |
| What kind of expertise becomes engaged | 1 |
| Who reported incident | 1 |
